# Implementation of Medication Disposal Programs and Availability of Same-Day Naloxone at Community Pharmacies: Protocol for a Secret Shopper Caller Approach

**DOI:** 10.2196/64344

**Published:** 2025-06-10

**Authors:** Kathleen L Egan, Briana Lewis, Kayleigh Fields, James McMillian III, Rachel L Graves, David M Kline, Lori Ann Eldridge

**Affiliations:** 1 Department of Implementation Science Division of Public Health Sciences Wake Forest University School of Medicine Winston Salem, NC United States; 2 Department of Health Education and Promotion College of Health and Human Performance East Carolina University Greenville, NC United States; 3 Department of Human Development and Family Sciences College of Health and Human Performance East Carolina University Greenville, NC United States; 4 Department of Emergency Medicine Wake Forest University School of Medicine Winston Salem, NC United States; 5 Department of Biostatistics and Data Science Division of Public Health Sciences Wake Forest University School of Medicine Winston Salem, NC United States

**Keywords:** opioid, naloxone, disposal, mystery caller, pharmacy, protocol, naloxone availability, harm reduction, opioid disposal, medication disposal, secret shopper

## Abstract

**Background:**

Pharmacies can implement multiple strategies, including medication disposal programs (eg, disposal boxes, deactivation products, and mail-back envelopes) and offering over-the-counter naloxone, to prevent nonmedical opioid use and overdose. The quantity of opioid prescriptions dispensed in the United States is so high that every other adult could receive one opioid prescription per year. Many of these opioids go unused and are kept in homes rather than disposed of after ceasing use. The primary source of prescription opioids for nonmedical use is relatives or friends, which suggests that the diversion of excess and retained prescription opioids contributes significantly to nonmedical use. Naloxone is a life-saving medication that works as an opioid antagonist to reverse the effects of opioids and restore normal breathing to a person experiencing an overdose. All 50 US states have passed laws (eg, statewide standing orders) that allow pharmacists to distribute naloxone without an individual patient prescription, and the US Food and Drug Administration approved the first over-the-counter naloxone medication in March 2023. Individual and neighborhood characteristics are associated with nonmedical opioid use and overdose. It is essential to ensure that pharmacy-based overdose prevention practices are widely available to all individuals.

**Objective:**

*:* This study aims to assess the extent to which disposal programs and same-day naloxone have been implemented in pharmacies across the United States and examine neighborhood characteristics in implementation. We hypothesize that as neighborhood disadvantage and the proportion of Black or African American residents in a neighborhood increase, the likelihood of a pharmacy having a disposal program or same-day naloxone decreases. We also hypothesize differences in medication disposal programs and same-day naloxone availability by retailer chain and type of pharmacy.

**Methods:**

A secret shopper caller protocol will be used to identify pharmacies that have implemented a medication disposal program and have naloxone available on the same day without a prescription. We will conduct disproportionate stratified random sampling with the strata being pharmacy chains to maximize the likelihood of sampling corporations and independent pharmacies. The goal is to obtain a final sample of 1000 pharmacies. Neighborhood characteristics will be appended to the secret shopper data. To explore neighborhood and pharmacy characteristics associated with the availability of medication disposal programs and same-day naloxone, we will use logistic regression. This protocol represents the entire structure of the secret shopper caller approach.

**Results:**

Data collection was completed in the spring of 2024. The expected results will be published in 2025.

**Conclusions:**

This will be the first study to examine national estimates of medication disposal programs, same-day naloxone availability at pharmacies, and the geographic characteristics associated with their implementation.

**International Registered Report Identifier (IRRID):**

DERR1-10.2196/64344

## Introduction

### Overdose Prevention at Community Pharmacies

Community pharmacists are essential members of frontline patient care. Their role in the overdose epidemic is critical in the defense against substance use disorder and opioid overdose [1,2]. Pharmacists are trained about substance use disorder during their educational instruction and can enhance their knowledge through continuing education on overdose prevention and opioid use disorder interventions [3]. It has been reported that the majority of pharmacists feel comfortable consulting about overdose prevention methods [4]. To address the opioid crisis, pharmacies have participated in prescription drug monitoring programs, implemented medication disposal programs, and dispensed naloxone and other medications for opioid use disorder [1].

### Medication Disposal Programs

Despite efforts in the United States to reduce the number of opioid prescriptions through prescribing guidelines [5,6] and drug monitoring programs [7,8], about 39.5 opioid prescriptions were dispensed per 100 persons in 2022 [9]. Many opioid medications go unused; a meta-analysis of postoperative opioid consumption for acute pain by US adults found that 61% [10] of medications remain after treatment. Among individuals who use prescription opioids inappropriately, most obtain them from family or friends, with or without their knowledge [11]. Thus, medication diversion is the primary source of prescription opioids for misuse. Facilitating the secure disposal of opioid medication during and after treatment is needed to prevent medication diversion.

The US Food and Drug Administration (FDA) endorses multiple methods to dispose of unused prescription opioids when they are no longer needed, including take-back days, disposal boxes, mail-back programs, and deactivation kits [12,13]. As of 2014, pharmacies have been able to offer these services to their customers [14]. Two studies examined the availability of medication disposal boxes in Kentucky and North Carolina [15,16]. In Kentucky, 144 medication disposal boxes had been implemented in community pharmacies as of 2020, with about 50% of counties having at least one box [16]. While rural counties in Kentucky had higher opioid dispensing rates than urban counties, they were less likely to have a medication disposal box [16]. In North Carolina, 350 (13.5%) pharmacies had implemented a medication disposal box as of 2021 [15]. The only study of national estimates of medication disposal box availability was conducted by the US Government Accountability Office in 2017. This assessment examined the list of authorized collectors from the US Drug Enforcement Administration (DEA) and found that 3% of pharmacies and other entities eligible to collect unused prescription drugs had registered with the DEA to implement a disposal box. A limitation of this study is that it relied on the DEA’s list of authorized collectors, which is not a reliable indicator of active medication disposal boxes [15,17]. To our knowledge, no studies have examined pharmacy participation in the distribution of mail-back envelopes or deactivation kits.

Broad access to medication disposal programs has been linked to reductions in opioid-related overdose deaths. One study found that communities with drug take-back programs had a decline in opioid overdose deaths, highlighting the role of these programs in limiting the availability of prescription opioids for misuse [18]. Various state and local studies have found a positive relationship between the number of medication disposal locations and decreased prescription drug misuse [19-21]. For instance, a study conducted in New York State demonstrated that increased drug take-back locations were associated with a reduction in prescription opioid misuse [22]. These findings underscore the importance of increasing access to safe disposal options as part of broader strategies to combat opioid misuse. Further studies are needed to establish the full impact on population-level outcomes related to misuse and overdose.

### Same-Day Access to Naloxone at Pharmacies

In 2023, the number of opioid-related drug overdoses in the United States decreased, rather than rose, for the first time since 2018 [23]. While there was a decrease in opioid-related deaths from 2022 to 2023, the number of deaths in 2023 (n=81,083) [24] was significantly higher than in 2018 (n=46,802) [25]. The provisional Centers for Disease Control and Prevention (CDC) data revealed that the decrease in opioid-related deaths was uneven across demographic groups, with Black individuals experiencing only a 6% reduction, Hispanic individuals having a 3% decrease, and American Indian or Alaska Native populations seeing a 2% increase. Naloxone is an opioid antagonist that is available as a nasal spray or injection [26]. Naloxone has been identified as a critical tool in combating the overdose epidemic in the United States [27,28]. During the last two decades, naloxone access has progressed from requiring a prescription to a standing order and recently to over-the-counter availability [29]. Studies that examined naloxone laws (layperson access) and opioid mortality have found that a decrease in opioid mortality occurs when naloxone is more accessible [30-32].

In 2023, the FDA approved the nasal spray naloxone for over-the-counter status [29]. This action theoretically should improve access to naloxone nationwide and will eliminate the need for a standing order or even a prescription. Previous studies that focused on naloxone availability with a standing order found that independent pharmacies, compared with chain pharmacies, were less likely to have same-day availability of naloxone [33-37]. Speculation has occurred that the status change of naloxone from prescription to over-the-counter may cause an increase in the price, thus adding a barrier to access [38,39]. Studies before the status change found that the out-of-pocket cost of naloxone varied greatly, with independent pharmacies charging more than community pharmacies [40,41].

### Socioeconomic Disparities

There are existing individual and neighborhood differences associated with nonmedical opioid use and related harms [42]. A systematic review found that 34 out of 37 studies published between 2000 and 2018 identified at least one characteristic linked to opioid overdose. The studies indicated that individual and neighborhood disadvantages, such as neighborhood-level poverty rates, median household income, county-level unemployment rates, neighborhood-level educational attainment, neighborhood-level dilapidated housing structures, and population-level criminal justice system involvement, were associated with higher opioid overdose rates [42]. Researchers have also identified socioeconomic and racial or ethnic differences in both the distribution of pharmacies [43-45] and the dispensing of opioid analgesics [46-48]. When individuals are not able to manage their pain with prescribed opioids, they may resort to diverting medications or the use of alternative sources of opioids (fentanyl and heroin) [49]. These differences may hinder the adoption of diversion programs and overdose prevention efforts. Pharmacy-based overdose prevention practices must be widely available to all individuals to not exacerbate documented differences.

### Objective

The overall objective is to assess the extent to which disposal programs and same-day naloxone have been implemented in pharmacies across the United States and examine place-based health disparities in implementation. Based on previous research [45,46,50-52], we hypothesize that as neighborhood disadvantage (hyp_1_) and the proportion of Black or African American residents in a neighborhood (hyp_2_) increase, the likelihood of a pharmacy having a disposal box or same-day naloxone decreases. We also hypothesize differences by retailer chain (hyp_3_) and type of pharmacy (ie, large chain, midsize chain, or independent; hyp_4_), following previous research indicating large differences in how pharmacy chains implement underage tobacco sales policies [53] and disposal programs in North Carolina [33].

## Methods

### Overview

This study uses a secret shopper caller protocol design and will be conducted at Wake Forest University School of Medicine and East Carolina University.

### Participants

#### Eligibility

Pharmacies are eligible to be included in the study if they are listed on the 2023 list of active licensed pharmacies from Hayes Directories, Inc and have a physical location. Nonretail, compounding, and veterinarian pharmacies will be excluded.

#### Sample Size

The sample size is 1000 pharmacies from across the United States.

#### Sample

We have obtained the most recent list (October 2023) of active licensed pharmacies from Hayes Directories, Inc. The Hayes list provides the name, phone number, fax, street address, and if it is part of a chain (ie, 8 or more locations) of retail community pharmacies (N=56,451). We will conduct a disproportionate stratified random sampling with the strata being pharmacy chains to maximize the likelihood of sampling diverse corporations and independent pharmacies. We will classify pharmacies into corporate chains with over 1000 store locations in the Hayes Directories, Inc list, including subsidiaries and brands (eg, some Walgreens operate as Duane Reade). We will identify eight strata based on the number of pharmacies in our database: (1) CVS, (2) Walgreens, (3) Walmart, (4) Rite Aid, (5) Kroger, (6) Albertsons, (7) Publix, and (8) other retail pharmacies. With 8 strata, 125 pharmacies per stratum will allow us to reach our goal of 1000 pharmacies across the United States. We will oversample 25 stores per stratum in case there is a need for replacement, for a total of 1200 pharmacies.

### Data Collection and Measures

#### Procedure

A total of 3 data collectors, “secret shoppers,” will be trained to call pharmacies posing as individuals who either need to dispose of medicine or obtain naloxone. The secret shoppers consist of one graduate student and two upper-level, 4th-year undergraduate students. Calls will be made rather than in-person store visits so that it is feasible to assess a sample of pharmacies across the nation. This also reflects the real-life scenario of community members calling pharmacies to determine available services before they visit the pharmacy. Secret shoppers will use a semistructured script when asking pharmacies about disposal programs and same-day naloxone. Training of the secret shoppers will consist of a 2-hour session in which data collectors will learn the script, make practice calls to pharmacies not within the sampling frame (up to 10 pharmacies per data collector), and agree on standard responses to potential questions. Separate calls will be made to query the availability of disposal programs and same-day naloxone. During and after each call, data collectors will fill out a secure REDCap (Research Electronic Data Capture; Vanderbilt University) [54,55] survey about their experience with the pharmacy staff they corresponded with on the call. The codebooks for the medication disposal program (Multimedia Appendix 1) and same-day naloxone without a prescription (Multimedia Appendix 2) have been included.

#### Primary Outcomes

There are two primary outcomes: (1) availability of a medication disposal box and (2) same-day naloxone. The two primary outcomes are distinct and do not occur within the same phone call. Each outcome represents a separate call type, addressing different aspects of the study. This will allow the secret shopper to capture the nuanced dynamics of the study’s focus more accurately. The availability of a medication disposal box will be assessed with the following question posed by a secret shopper: “Hi, I have some Vicodin that I need to get rid of. I heard some pharmacies will take back medications or give you something to dispose of them at your home. Does your pharmacy do this?” The secret shopper will code if the pharmacist responds, “Yes, disposal box.” Other potential options that can be recorded include “no”; “yes, give it directly to the pharmacist”; “yes, other option”; and “yes, but no additional information was provided.” To assess same-day availability of naloxone without a prescription, the caller will ask, “Hi, I am calling to find out if I can get Narcan today?” and “Do I need a prescription?” If the pharmacist states that they have naloxone available that day and that a prescription was not needed, the pharmacy will be coded as having same-day naloxone without a prescription.

#### Other Measures

Using the secret shopper caller protocol, we will also collect data on where the medication disposal box and naloxone are located in the store, where they could dispose of their medication if the pharmacy would not take it back, where else they could get naloxone, and the cost of naloxone.

We will obtain neighborhood characteristics at the census block group and tract level for the sampled pharmacies from Social Explorer (a subscription service for census and other data) and the University of Wisconsin’s Neighborhood Atlas (Table 1). We will use data from the 2022 (most recent) five-year estimates from the US Census Bureau’s American Community Survey, a multiyear probability-based estimate suitable for use with small area units. The Neighborhood Atlas’s Area Deprivation Index is publicly available and created at the block group level, providing a more localized measure of neighborhood disadvantage. Rurality will be defined based on the US Department of Agriculture’s Rural-Urban Commuting Area Codes [56]. We will obtain county-level drug overdose rates for the most recent year available from the CDC’s National Vital Statistics System.

**Table 1:** Measures and data sources.

Variable (type or form)				Source	Level
**Outcome**					
	Availability of a medication disposal box (yes vs no)		Secret Shopper		Store
	Availability of same-day naloxone without a prescription (yes vs no)		Secret Shopper		Store
**Predictors and covariates**					
	**Pharmacy characteristics**				
		Corporate owner (top 7 pharmacy chains by number of stores exceeding 1000)	Hayes		Store
		Type (chain, food, independent, and medical)	Hayes		Store
	**Neighborhood resources—Bernard framework’s economic domain**				
		Area Deprivation Index	University of Wisconsin Neighborhood Atlas		Block group
		Percentage of the population living under the federal poverty line (ratio scaled ×10, eg, 12%=1.2)	ACS^a^		Tract
		Percentage of the population age ≥16 years that is unemployed (ratio scaled ×10, eg, 12%=1.2)	ACS		Tract
	**Neighborhood structure—Bernard framework’s physical domain**				
		Area Deprivation Index	University of Wisconsin Neighborhood Atlas		Block group
		Percentage of housing structures that are vacant (ratio, scaled ×10, eg, 12%=1.2)	ACS		Tract
		Percentage of housing structures that are renter occupied (ratio scaled ×10, eg, 12%=1.2)	ACS		Tract
		Rurality or urbanicity (rural-urban commuting area codes)	USDA^b^		Tract
	**Neighborhood composition—Bernard framework’s institutional domain**				
		Percentage of the population identifying American Indian or Alaska Native as their race (ratio, scaled ×10, eg, 12%=1.2)	ACS		Tract
		Percentage of the population identifying Black or African American as their race (ratio, scaled ×10, eg, 12%=1.2)	ACS		Tract
		Percentage of the population identifying Latino or Hispanic as their ethnicity, of any race (ratio, scaled ×10, eg, 12%=1.2)	ACS		Tract
		Percentage of the population identifying White as their sole race, of any ethnicity (ratio, scaled ×10, eg, 12%=1.2)	ACS		Tract
	**County overdose deaths**				
		County-level drug overdose deaths	CDC’s^c^ National Vital Statistics System		County

^a^ACS: American Community Survey.

^b^USDA: US Department of Agriculture.

^c^CDC: Centers for Disease Control and Prevention.

### Data Management and Ethics

We will implement protections to limit any ability to link data with individuals. We will not collect information that identifies the pharmacists or pharmacy staff whom we speak to on the calls. The only identifiable information will be the pharmacies in our sample. To minimize the likelihood of a breach in confidentiality, data will be collected and stored in REDCap, a secure web application for building and managing surveys, and on a secure and encrypted storage system maintained by Wake Forest University School of Medicine IT Security. Public access to deidentified data will be made public after the study has concluded.

### Data Analysis

#### Geocoding and Spatial Data

After address cleaning and standardization, we will use Environmental Systems Research Institute, Inc’s geocoding service to geocode drop box locations and use the resulting latitude and longitude to “spatially join” pharmacy locations with a shapefile of census geographies to obtain a Federal Information Processing Standards code in ArcGIS Desktop 10.6.1 (Esri). The Federal Information Processing Standards code will allow us to link data to standard area units such as block groups, tracts, and counties in a single dataset and provide a unique ID for each level of nested area units.

#### Statistical Analyses

##### Analysis for Availability Estimates

To provide weighted estimates based on the availability of a medication disposal box and same-day naloxone nationally and for each stratum, weighing will be based on the percentage of corporate pharmacies in our sample compared with the total population. Using procedures that account for the stratified survey design, we will estimate the proportion and 95% CI to describe availability nationally and in each stratum for each outcome. Analyses will be conducted using SAS 9.4 (SAS Institute).

##### Analysis for Logistic Regression Models

To explore neighborhood and pharmacy characteristics associated with availability, we will use logistic regression with robust SEs where the availability of medication disposal boxes and same-day naloxone without a prescription are the outcomes. The stratified survey design will be accounted for when fitting the logistic regression model. Robust SEs will be used to account for potential correlation due to pharmacies being in the same state. We will initially fit unadjusted models for each characteristic of interest and then will fit multivariable models that include characteristics of interest and any available potential confounders for the associations of interest. Analyses will be conducted using SAS 9.4. Estimates will be presented as odds ratios with 95% CIs.

#### Statistical Power

##### Power for Availability Estimates

With 8 strata in a stratified random sample, a sample of 1000 should provide a relative SE of 11.7% for the population estimate (ie, we would expect to be 95% certain that the national estimate was within ±3.1 percentage points of the estimate). Individual strata would have an average relative SE of 25% and <±10 percentage points for each stratum.

##### Power for Logistic Regression Models

The total sample size (N=1000) was determined by a logistic regression power analysis. Initial parameter estimates in the Monte Carlo simulations (using MPlus 8; Muthén and Muthén) were based on a previous study of medication disposal programs at pharmacies in North Carolina [15]. The unconditional and conditional models achieved power of over 0.80 with two-tailed α=.05. The minimum detectable effect size was estimated at level 1 to be 0.17 with a small, medium, or large intraclass correlation coefficient (ICC) value and was estimated at level 2 to be 0.48 for a small ICC value, 0.50 for a medium ICC value, and 0.43 for a large ICC value [57].

### Ethical Considerations

Informed consent was waived for this study. The consent process would reveal the secret shopper caller protocol and potentially result in the collection of inaccurate data [58]. Data will not be collected or recorded in a manner that would identify human participants. This protocol has been approved by the Wake Forest University School of Medicine Institutional Review Board (00105728).

## Results

Data for this study were collected from January to April 2024. As of April 2024, the sample consists of 1108 pharmacies. Expected publication of the results is the summer of 2025.

## Discussion

### Contributions to the Literature

The overall objective of this study is to assess the extent to which disposal programs and same-day naloxone have been implemented in pharmacies across the United States and examine neighborhood characteristics related to their implementation. Our central hypothesis is that same-day naloxone will be more readily available at chain pharmacies than at independent pharmacies. The availability of medication disposal programs and same-day naloxone will be assessed using a secret shopper approach. Furthermore, we hypothesize that as neighborhood disadvantage and the proportion of Black and African American residents in a neighborhood increase, the likelihood of a pharmacy having a disposal program or same-day naloxone will decrease.

### Next Steps

As part of the larger study, following the completion of the secret shopper caller study, interviews will be conducted with a sample of 60 pharmacy managers whose pharmacies were assessed in the secret shopper study. The objective of these interviews is to develop preliminary approaches grounded in implementation science frameworks to improve the implementation of medication disposal programs and same-day naloxone at community pharmacies.

### Use of a Secret Shopper Protocol to Assess Public Health Interventions at Pharmacies

The use of a secret shopper caller protocol allows for unbiased and direct observations of overdose prevention service quality and compliance within community pharmacies [58]. An evaluation of how well pharmacy staff adhere to protocols, policies, and laws regarding overdose prevention can be determined. Similar approaches have also been used to assess other public health interventions at community pharmacies [59]. This method allows for real-time feedback and identification of areas needing improvement, ensuring that interventions are delivered effectively and consistently.

### Limitations

There are several limitations to the secret shopper study. One limitation is that callers only speak with one pharmacy staff member, who may not represent all pharmacy staff. For this study, we documented whether the staff member revealed their role in the pharmacy and, if so, what their role was. To limit uncertainty, a future study could add a specific question in the secret shopper script to confirm whether the interaction was with a pharmacist. Another potential limitation is that the secret shoppers will only speak English during interactions, which restricts the findings to English-speaking populations. Future research could address this limitation by incorporating multilingual secret shoppers. We expect that additional limitations may be identified during study completion, and these will be identified and described in the results-oriented paper upon completion of the study.

### Conclusion

Upon successful completion of the study, we will have estimates of the availability of disposal programs and same-day naloxone at pharmacies across the United States and identify place-based health disparities associated with their implementation.

## Appendix

Multimedia Appendix 1 Mystery caller data collection form for medication disposal programs.

Multimedia Appendix 2 Mystery caller data collection form for naloxone.

## Abbreviations

CDC: Centers for Disease Control and Prevention
DEA: Drug Enforcement Administration
FDA: Food and Drug Administration
ICC: intraclass correlation coefficient
REDCap: Research Electronic Data Capture

## Data Availability

The datasets generated or analyzed during this study are available from the corresponding author on reasonable request.
