# Supplementary material for: Implementation of Medication Disposal Programs and Availability of Same-Day Naloxone at Community Pharmacies: Protocol for a Secret Shopper Caller Approach
Source: JMIR Res Protoc. 2025 Jun 10;14:e64344. doi: 10.2196/64344 (PMC12188141; doi:10.2196/64344)
Supplement: Multimedia Appendix 2 [file resprot_v14i1e64344_app2.pdf]

Naloxone Mystery Caller Data Collection

Record ID ______________________

Record ID ______________________

INSTRUCTIONS:

Before you make the call -

1) Add the pharmacy ID number from the Excel Spreadsheet.
2) Add the phone number associated with the pharmacy ID from the Excel Spreadsheet.
3) Add your mystery caller ID.

Pharmacy ID ______________________

Phone Number ______________________

Mystery Caller ID ______________________

INSTRUCTIONS:

Now, you are ready to make the call. A few notes before you dial -

*Wait at least 1 minute for someone to answer your call. If no one answers after 1 minute, end the attempt.
*If they put you on hold, end the attempt after 4 minutes of waiting.
*If they offer to call you back, politely decline.

Make sure to record details at about your attempted call towards the end of this data collection form. We will make up to three attempts for each pharmacy on different days.

About your role: You are someone who lives with a person who is at risk for overdose and you want to ensure that you have Narcan in case they overdose

"Hi, I am calling to find out if I can get Narcan today?"

- ◯ No, not today
- ◯ No, we do not sell naloxone
- ◯ Yes
- ◯ Other

If they provided a response that was not an option for you, summarize the response here. ______________________

"Do I need a prescription?"

- ◯ No
- ◯ Yes
- ◯ Other

Specify other ______________________

"About how much does Narcan cost?"<br><br>(don't include $ sign)<br><br>If they ask about insurance, tell them that you don't have your insurance information on you and that you cannot remember the provider.	______________________
"Where is Narcan located in the store?"	◯ Pharmacy counter<br>◯ Front check out<br>◯ Other
Where is the 'other' location?	______________________
"Ok. Do you know where I should go to get Narcan?"<br><br>Did the staff member indicate other ways (other than their pharmacy) that naloxone could be accessed?	◯ No<br>◯ Yes
Where did they suggest you get Narcan?	◯ Another pharmacy<br>◯ Public Health Department<br>◯ Syringe Service Program (SSP)<br>◯ Other
Specify other places to get naloxone	______________________
Please include any other noteworthy questions or statements made by the pharmacist/pharmacy staff.	______________________
Did the pharmacy staff member reveal their current position?	◯ Yes<br>◯ No
What was their role?	◯ Pharmacist<br>◯ Pharmacy Assistant<br>◯ Pharmacy Technician<br>◯ Other
INSTRUCTIONS:<br><br>The remaining section is to record information about your call attempt(s).	
Date of Call (1)	______________________<br>(MM/DD/YYYY)
Time of Call (1)	______________________<br>(HH:MM (am/pm))
Were you able to speak with a pharmacy staff member about naloxone? (1)	◯ No<br>◯ Yes<br>(no--> end call)

Date of Call (2)	__________________________________<br>(MM/DD/YYYY)
Time of Call (2)	__________________________________<br>(HH:MM (am/pm))
Were you able to speak with a pharmacy staff member about naloxone? (2)	◯ No<br>◯ Yes<br>(no--> end call)
Date of Call (3)	__________________________________<br>(MM/DD/YYYY)
Time of Call (3)	__________________________________<br>(HH:MM (am/pm))
Were you able to speak with a pharmacy staff member about naloxone? (3)	◯ No<br>◯ Yes<br>(no--> end call)
Was this pharmacy still open for business (i.e, not closed for good)?	◯ Yes<br>◯ No
Does this pharmacy serve community members (assume yes unless they tell you otherwise)?	◯ Yes<br>◯ No
